# Functional Comparison between Genes Dysregulated in Ulcerative Colitis and Colorectal Carcinoma

**DOI:** 10.1371/journal.pone.0071989

**Published:** 2013-08-22

**Authors:** Wenyuan Zhao, Lishuang Qi, Yao Qin, Hongwei Wang, Beibei Chen, Ruiping Wang, Yunyan Gu, Chunyang Liu, Chenguang Wang, Zheng Guo

**Affiliations:** 1 College of Bioinformatics Science and Technology, Harbin Medical University, Harbin, China; 2 Department of Bioinformatics, School of Basic Medical Sciences, Fujian Medical University, Fuzhou, China; INSERM, France

## Abstract

**Background:**

Patients with ulcerative colitis (UC) are predisposed to colitis-associated colorectal cancer (CAC). However, the transcriptional mechanism of the transformation from UC to CAC is not fully understood.

**Methodology:**

Firstly, we showed that CAC and non-UC-associated CRC were very similar in gene expression. Secondly, based on multiple datasets for UC and CRC, we extracted differentially expressed (DE) genes in UC and CRC versus normal controls, respectively. Thirdly, we compared the dysregulation directions (upregulation or downregulation) between DE genes of UC and CRC in CRC-related functions overrepresented with the DE genes of CRC, and proposed a regulatory model to explain the CRC-like dysregulation of genes in UC. A case study for “positive regulation of immune system process” was done to reveal the functional implication of DE genes with reversal dysregulations in these two diseases.

**Principal Findings:**

In all the 44 detected CRC-related functions except for “viral transcription”, the dysregulation directions of DE genes in UC were significantly similar with their counterparts in CRC, and such CRC-like dysregulation in UC could be regulated by transcription factors affected by pro-inflammatory stimuli for colitis. A small portion of genes in each CRC-related function were dysregulated in opposite directions in the two diseases. The case study showed that genes related to humoral immunity specifically expressed in B cells tended to be upregulated in UC but downregulated in CRC.

**Conclusions:**

The CRC-like dysregulation of genes in CRC-related functions in UC patients provides hints for understanding the transcriptional basis for UC to CRC transition. A small portion of genes with distinct dysregulation directions in each of the CRC-related functions in the two diseases implicate that their reversal dysregulations might be critical for UC to CRC transition. The cases study indicates that the humoral immune response might be inhibited during the transformation from UC to CRC.

## Introduction

Ulcerative colitis (UC) is a chronic inflammatory disease caused by persistent pro-inflammatory stimuli, such as microbial products and autoimmune injury [1], and patients with UC are at increased risk for development of colorectal cancer (CRC), specifically referred to as colitis-associated colorectal cancer (CAC) [2]. Current evidence suggests that molecular alterations in patients with UC may promote neoplastic transformation by disturbing multiple cancer-related functions. For example, it has been found that pro-inflammatory factors released from the innate and adaptive immune systems in UC patients, such as nuclear factor (NF)-κB [3], interleukin (IL)-6 [4], and tumor necrosis factor-alpha [5], contribute to the development of colon neoplasia by stimulating proliferation and angiogenesis [6], [7], and by promoting DNA damage to the intestinal epithelium cells [8]. As alterations in gene expression largely reflect the functional disorders [9], it is possible that genes involved in some cancer-related functions could have similar expression dysregulations in UC with those in CAC. On the other hand, it is known that the disturbance in some critical cancer-related functions occurs in opposite directions in UC and CAC. For examples, it has been found that Fas-mediated apoptosis is promoted in UC [10] but inhibited in CAC [11] and that some key pro-inflammatory mediators, such as IL-13, upregulated in UC may contribute to the host defense against malignancy [12], [13]. Therefore, identifying genes with distinct dysregulations in UC and CAC might provide hints for understanding their critical roles in the transformation from UC to CAC. As a specific type of CRC, CAC evolves through the inflammation–dysplasia–carcinoma sequence, different from another type of CRC, non-UC-associated CRC, that evolves through the adenoma–carcinoma sequence CRC [14]. On the other hand, UC-associated and non-UC-associated CRC could be very similar in gene expression, as non-UC-associated CRC is also somehow driven by pathways involved in inflammation [15] with similar inflammatory signature genes characteristic of CAC [16] and non-UC-associated CRC and CAC are similar in most of the essential stages of cancer development with similarly disturbed signaling pathways [17].

Previous studies investigating the mechanism of the transformation from UC to CAC have mainly focused on a few candidate genes or pathways using animal models [5], [7]. Recently, based on high-throughput microarrays, some studies have compared the genome-wide expression profiles between UC and CRC (both CAC and non-UC-associated CRC), focusing on identifying genes that are differentially expressed between the two diseases mainly for the purposes of distinguishing CRC from UC [18], [19]. However, an individual gene can be detected to be differentially expressed between two diseases in different situations; for instance, the gene may be dysregulated in only one of the diseases, but not in the other, or it may be dysregulated in both diseases, in either the same or the opposite direction (i.e. upregulated versus downregulated cases shown in Figure S1). Thus, directly comparing the expression profiles between two diseases cannot discriminate the sources of the difference and thus may miss critical information about the transcriptional relationship between the two diseases. Therefore, it would be interesting to compare the expression profiles between UC and CAC taking the normal controls as the background. Notably, current microarray data for CRC usually do not include explicit information about whether the patients with CRC are developed from UC, possibly due to the difficulty in determining such information for CRC patients. Nevertheless, as UC-associated and non-UC-associated CRC could be very similar in gene expression, comparing UC with CRC including both UC-associated and non-UC-associated CRC could still provide hints for understanding the transformation from UC to CAC. It would be reasonable to assume that non-UC-associated CRC would be unlikely to be more similar to UC than CAC. Thus, if we can find a similar dysregulation pattern between UC and CRC with mixed subtypes, the similarity would be kept between UC and CAC, although there would be some uncertainties for interpreting the observation on the transcriptional difference between UC and CRC with mixed subtypes.

In this study, using a dataset consisting of gene expression profiles of both CAC and non-UC-associated CRC, we firstly showed that UC-associated and non-UC-associated CRC were very similar in gene expression. Secondly, based on multiple microarray datasets for each disease, we extracted differentially expressed (DE) genes in patients with UC and CRC versus normal controls, respectively. Thirdly, we assessed the functions overrepresented with the DE genes of CRC, termed CRC-related functions, and showed that the dysregulation directions of the DE genes of UC were significantly similar to the findings for CRC in these CRC-related functions. Fourthly, we proposed a regulatory model to explain the CRC-like dysregulation of genes in UC, based on the assumption that the dysregulation of the genes in UC could be regulated by transcription factors (TFs) that are affected by pro-inflammatory stimuli for colitis. Finally, a case analysis for a CRC-related function, “positive regulation of immune system process”, was done to reveal the functional implication of the small portion of genes with the opposite dysregulation directions in the two diseases for the transformation from UC to CRC.

## Materials and Methods

### Microarray Data

The dataset GSE3629 was collected from the NCBI Gene Expression Omnibus (GEO; http://www.ncbi.nlm.nih.gov/geo/ [20]), which contained 62 non-UC-associated CRC samples, 6 CAC samples and 43 UC samples. Unfortunately, this dataset contained no normal controls, so it is not suitable for comparing the gene expression of UC and CAC taking the normal controls as the background. We used this dataset to compare the expression profiles of non-UC-associated CRC and CAC taking the UC as the background.

Three UC datasets and four CRC datasets generated by the Affymetrix Human Genome U133 Plus 2.0 Array were also collected from the NCBI Gene Expression Omnibus (GEO; http://www.ncbi.nlm.nih.gov/geo/ [20]), as described in Table 1. The raw mRNA expression data was preprocessed using the Robust Multi-array Average (RMA) algorithm [21]. We used the SOURCE database [22] to map the ProbeIDs into GeneIDs. As no dataset with explicit information of stage I CRC samples was available, we analyzed these datasets including stage I-III CRC samples, based on the assumption that stage II and III CRC samples would be unlikely to be more similar to UC than stage I samples. The logic behind this assumption is that if we can find a similar dysregulation pattern between UC and CRC in different stages, then this similarity should be kept between UC and early UC-associated CRC.

**Table 1:** The microarray datasets analyzed in this study.

Accession id	Disease type	Sample size (Disease VS Normal)
GSE16879	UC	24∶ 12
GSE10191	UC	8∶ 11
GSE10616	UC	10∶ 11
GSE9348	CRC	70∶ 12
GSE18105	CRC	17∶ 17
GSE20916	CRC	36∶ 24
GSE23878	CRC	35∶ 24

**Notes:** Patients with CRC from GSE9348 were at an early stage (Stage I/II), patients with CRC from GSE18105 were at stage II and stage III, and patients with CRC from GSE23878 and GSE20916 were metastasis-negative. In the datasets GSE18105, we just used the 17 paired CRC and adjacent normal samples to assure the clinical characteristics matching.

### Genes Participating in Immune Response

The Immunome database (http://bioinf.uta.fi/Immunome/) [23] contains nine categories of immune genes, including humoral immunity, cellular immunity, inflammation, and chemokines and their receptors. Based on this database, we analyzed the distribution of the DE genes whose dysregulation occurred in opposite directions in UC and CRC in various immune responses. Using the Immune Response in Silico database (IRIS) [24], we analyzed the distribution of the DE genes with opposite dysregulation directions specifically expressed in various immune cells, including T cells, B cells, natural killer cells and dendritic cells.

### Selecting DE Genes from Multiple Datasets for a Disease

We created a DE gene list from multiple datasets for each disease, as a dataset can usually capture only a certain number of the DE genes, owing to small sample sizes, large biological variations and technical limitations [25], [26]. Firstly, we selected DE genes from each dataset using the Significance Analysis of Microarrays (SAM) method [27] at a false discovery rate (FDR) control level of 1%. Then, we evaluated the reliability of DE genes extracted from different datasets for each disease by applying the binomial distribution model to evaluate the significance of the percentage of the DE genes with the same dysregulation directions in the DE genes commonly detected for every two datasets. Finally, we integrated the DE genes from all datasets for each disease according to the following criterion: DE genes selected in at least one dataset were included in the list, after excluding those DE genes in multiple datasets that had inconsistent dysregulation directions across these datasets.

### Finding CRC-related Functions

The Gene Ontology (GO) annotation data [28] were downloaded on October 1, 2011. We used the GO-function algorithm [29] to select GO Biological Process (GO BP) terms that are significantly enriched with CRC-related DE genes with FDR<1% as assessed by the Benjamini–Hochberg procedure [30], which we termed “CRC-related functions”. In this study, we used the GO-function algorithm to treat local redundancy [29]: when both an ancestor term and an offspring term are detected as statistically significant, the ancestor term will be extracted as biologically relevant if there is evidence to suggest that the remaining genes are still likely to be relevant to the disease after the removal of genes in its significant offspring term(s); otherwise, only the offspring term is kept [29].

### Transcriptional Regulatory Model

From the Transcription Factor Database (TRANSFAC) [31] and the Transcriptional Regulatory Element Database (TRED) [32], we collected 363 TFs together with their target genes. We defined the GO BP term “response to stimulus” and its offspring terms as the stimulus-related terms, and found the stimulus-related terms which significantly enriched with UC-related DE genes with FDR<1%, termed as “UC-stimulus-functions”.

If a DE TF in a UC-stimulus-function *F*_1_ can regulate a UC-CRC consistent DE gene in a CRC-related function *F*_2_ in UC, we defined them as a TF-target pair between *F*_1_ and *F*_2_. We assumed that if there were significantly more TF-target pairs between *F*_1_ and *F*_2_, then CRC-related function *F*_2_ could be regulated by UC-stimulus-function *F*_1_ associated with UC. We then calculated the statistical significance of the number of TF-target pairs between *F*_1_ and *F*_2,_ using the hypergeometric model as follows:
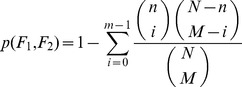
where *N* is the total number of TF-target pairs in the TRANSFAC and TRED databases; *M* is the number of the TF-target pairs between DE TFs and their DE target genes in UC; and *n* and *m* are the numbers of gene/DE TF-target pairs between TFs in *F*_1_ and their target genes in *F*_2_ after removing the overlapping genes between *F*_1_ and *F*_2_. The P values were adjusted using the Benjamini–Hochberg procedure [30].

## Results

### Similar DE Genes in UC and CRC

Firstly, using the SAM method with 10% FDR control, we found no genes differentially expressed between non-UC associated CRC and CAC based on the dataset GSE3629. In this dataset, with 10% FDR control, we selected 15174 DE genes between non-UC-associated CRC and UC and 12113 DE genes between CAC and UC. We found that 99.73% of the 10357 overlapped DE genes were consistent in dysregulation directions for non-UC-associated CRC and CAC taking the UC as the background, which was significantly higher than what expected by chance (binomial test, P<1×10^–16^). These results indicated that non-UC-associated CRC and CAC were highly similar in gene expression. Therefore, it would be largely reasonable to compare the gene expression between UC and CRC with mixed subtypes for studying the transitional mechanisms from UC to CAC.

Using the 1% FDR control, we extracted DE genes from each of the four datasets for CRC. Comparing any two of the four CRC datasets, as shown in Table 2, 98.47–99.73% of the DE genes commonly detected were consistent in dysregulation directions for both datasets, significantly higher than expected by chance (binomial test, all P<1×10^–16^). The non-random consistent signals between the DE gene lists extracted from independent datasets indicated that each of the DE gene lists was able to capture a portion of the effective biological signals associated with CRC [25], [26]. Therefore, we integrated the DE genes from all of the four CRC datasets (see Materials and Methods) and obtained a list of 11,038 CRC-related DE genes.

**Table 2:** The consistency of every two datasets for CRC.

Datasets	GSE9348	GSE18105	GSE20916	GSE23878
**GSE9348**	100% (7251/7251)*	98.54% (4061/4121)	99.73% (4761/4774)	98.84% (3226/3264)
**GSE18105**	98.54% (4061/4121)	100% (6041/6041)	98.57% (3730/3784)	98.47% (2635/2676)
**GSE20916**	99.73% (4761/4774)	98.57% (3730/3784)	100% (6679/6679)	99.19% (3077/3102)
**GSE23878**	98.84% (3226/3264)	98.47% (2635/2676)	99.19% (3077/3102)	100% (4504/4504)

**Notes:** *(number1/number2) followed the percentage of the DE genes with consistent dysregulation direction in all commonly detected DE genes between two datasets represent the number of the DE genes with consistent dysregulation direction and the number of all commonly detected DE genes, respectively.

**Figure 1:**
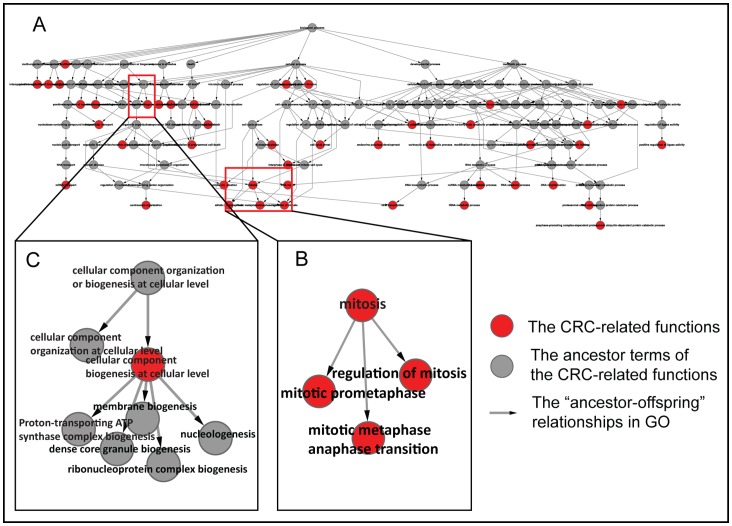
The CRC-related functions in the directed acyclic graph of Biological Process. **A.** All the CRC-related functions. **B.** A case for both the ancestor and offspring terms retained simultaneously. **C.** A case for just one term retained in a biological process branch.

Then, using the GO-function algorithm, with FDR control of 1%, we selected 44 CRC-related terms located in different branches of the directed acyclic graph of biological process, as shown in Figure 1A. Some of these CRC-related terms had ancestor-offspring relationships. For example, as shown in Figure 1B, the “mitosis” and its three offspring terms “mitotic prometaphase”, “mitotic metaphase/anaphase transition” and “regulation of mitosis” were retained simultaneously. It is known that the “mitosis” is related to cancer [33], whereas the three offspring terms are important steps in the mitosis [34]. In general, most of the 44 terms such as “apoptosis” and “cell proliferation” were well known cancer-associated functions, and many other terms could also be explained. For example, as shown in Figure 1C, the significant term “cellular component biogenesis at cellular level” includes “membrane biogenesis” and “nucleologenesis” as offspring terms which are necessary for mitosis whose dysregulation is related to the abnormal proliferation of cancer cells [35]. For another example, the significant term “macromolecule modification” includes “DNA modification”, “RNA modification”, “protein modification process” and “macromolecule methylation” as offspring terms, with the type of modification being, besides others, methylation such as DNA methylation or demethylation associated with carcinogenesis [36], [37]. Similarly, for the significant term “interspecies interaction between organisms”, accumulated evidences indicate that interaction between intestinal bacteria and host is significantly related with the occurrence and development of the CRC [38], [39].

Similarly, we selected a list of DE genes from each of the three datasets for UC. As shown in Table 3, 98.61–100% of the DE genes commonly detected in any two of the three UC datasets were consistent in the dysregulation direction for both datasets, significantly higher than expected by chance (binomial test, all P<1×10^–16^). We therefore obtained a list of 6,557 UC-related DE genes by integrating the DE genes from the three UC datasets (see Materials and Methods). These 6,557 UC-related DE genes overlapped with 4,764 of CRC-related DE genes, among which 72.33% had consistent dysregulation directions in the two diseases, significantly higher than expected by chance (binomial test, P<1×10^–16^). DE genes overlapping between UC and CRC with the consistent dysregulation direction in UC and CRC compared with the normal controls were termed “UC-CRC consistent DE genes”, whereas genes that had the reverse dysregulation directions in UC and CRC were termed “UC-CRC inconsistent DE genes”. In all of the 44 CRC-related functions, except for “viral transcription”, the frequencies of the UC-CRC consistent DE genes among the overlapping DE genes were >50%., and the frequencies were significantly higher than expected by random chance in 38 of these CRC-related functions (binomial test with random frequency set at 0.5, FDR<5%). The genes consistently and inconsistently dysregulated were listed in Table S1 and Table S2.

**Table 3:** The consistency of every two datasets for UC.

Datasets	GSE16879	GSE10191	GSE10616
**GSE16879**	100% (5041/5041)	98.61% (1634/1657)	98.93% (1382/1397)
**GSE10191**	98.61% (1634/1657)	100% (3171/3171)	100% (2327/2327)
**GSE10616**	98.93% (1382/1397)	100% (2327/2327)	100% (2367/2367)

The above results indicated that UC patients had CRC-like expression dysregulation patterns in most of the CRC-related functions. A potential explanation for this observation is that in patients with UC TFs dysregulated by pro-inflammatory stimuli could dysregulate genes in CRC-related functions, as analyzed in the following section.

### A Model for Pro-inflammatory Stimuli Regulating CRC-related Functions in UC

**Figure 2:**
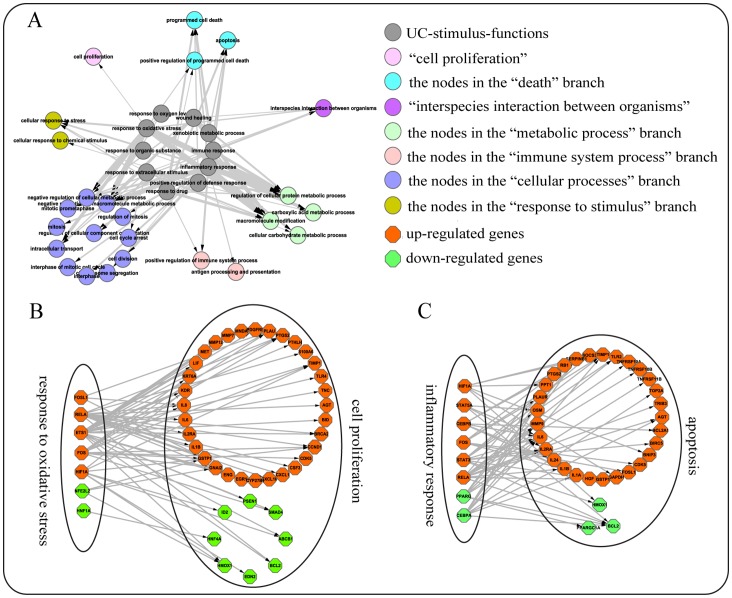
The significant regulatory links between UC-stimulus-functions and CRC-related functions in UC. **A.** The significant regulatory relationships between functions. The gray nodes represent the stimulus-related functions in UC, whereas the other nodes in each color represent functions that are located in the same branch of the Gene Ontology Biological Process (GO BP) tree. Edges represent the significant links between transcription factors (TFs) dysregulated in stimulus-related functions and their differentially expressed (DE) target genes in CRC-related functions in UC (see details in Materials and Methods), and its thickness is proportional to the significance level (-log_10_[P value]). **B.** A case for the significant regulatory links from “response to oxidative stress” to “cell proliferation”. **C.** Another case for the significant regulatory links from “inflammatory response” to “apoptosis”. These pink and green diamond nodes, in “response to oxidative stress” and “inflammatory response”, represent upregulated and downregulated DE genes in UC, respectively. In “cell proliferation” and “apoptosis”, the pink and green diamond nodes, respectively, represent genes consistently upregulated or downregulated in UC and CRC. An arrow represents the regulation relationship between a TF and one of its targets.

In the “response to stimulus” branch of the GO BP, using the GO-function algorithm with FDR control of 1%, we detected 14 UC-stimulus-functions, including “inflammatory response”, “response to extracellular stimulus”, “response to oxidative stress”, etc. Using the hypergeometric test with FDR<10%, we found that 25 CRC-related functions could be significantly regulated by dysregulated TFs in at least one of the UC-stimulus-functions (see Materials and Methods), as shown in Figure 2A. For example, as shown in Figure 2B, seven DE TFs dysregulated in the UC-stimulus-function “response to oxidative stress” could regulate 40 target genes, which were similarly dysregulated in both UC and CRC, in the CRC-associated function “cell proliferation”. Specifically, the TF ETS1 in “response to oxidative stress” was upregulated in UC, which could upregulate its target gene MET tyrosine kinase receptor to trigger mitogen-activated protein kinase (MAPK) cascades to promote cell proliferation in UC [40], in a similar way to CRC [41]. Similarly, the TF FOS was also upregulated in UC, and this could repress transcription of its target gene SMAD4 [42] to promote cell proliferation and lymph-node metastases [43], which again is similar to CRC [44]. These results indicated that “cell proliferation” could be accelerated by the dysregulated TF genes in the UC-stimulus-function “response to oxidative stress” to promote tumorigenesis.

For another example, as shown in Figure 2C, eight DE TFs dysregulated in the UC-stimulus-function “inflammatory response” could regulate 32 target genes, which were similarly dysregulated in both UC and CRC, in the CRC-associated function “apoptosis”. Specifically, IL-6 in the “inflammatory response” was upregulated in UC, which could enhance the proliferation of intestinal epithelial cells and increases their resistance to apoptosis in UC [6], meanwhile the proliferative and survival effects of IL-6 were mediated by STAT3 in colitis associated cancer [6]. However, despite many genes related with apoptosis were similarly dysregulated in both UC and CRC, the apoptosis is promoted in active UC but inhibited in colon cancer [10]. These results indicated that a few genes with different dysregulation patterns between UC and CRC may play key roles in determining the final apoptosis signals for the two diseases.

Similar analyses could be done for the other CRC-related functions. The transcriptional regulatory model could provide hints for analyzing the tumor-promoting effect of stimulus-related TFs in inflammatory environment. In fact, many of the DE TFs dysregulated in the UC-stimulus-functions have been reported to function as a tumor promoter in inflammation-associated cancer, including all of the seven TFs shown in Figure 2B, which were FOSL1 [45], ETS1 [46], FOS [47], HIF1A [48], RELA [49], NFE2L2 [50] and HNF1A [51]. Different from most of previous studies which focused on individual candidate TF, our study could provide a new angle to reveal the combination of several TFs in one UC-stimulus-function for dysregulating a CRC-related function in patients with UC which could contribute to the development from UC to CRC.

### UC-CRC Inconsistent DE Genes in the CRC-related Functions

For each of the CRC-related functions in which UC patients showed a significantly similar dysregulation pattern, there were also a certain percentage of genes with reverse dysregulations in the two diseases. In the “positive regulation of immune system process” of these 38 significantly consistent CRC-related functions, the frequency of the UC-CRC inconsistent DE genes was the highest (42.26%). Thus, we took it as a case to further analyze the UC-CRC inconsistent DE genes between UC and CRC.

Using the Immunome database, we found that the UC-CRC inconsistent DE genes in the “positive regulation of immune system process” were significantly enriched in the category of “humoral immunity” mediated by antibodies secreted by B lymphocytes (P = 0.048). Using the IRIS database, we also found that the UC-CRC inconsistent DE genes were enriched in genes specifically expressed in B cells (P = 0.016). All of the 22 UC-CRC inconsistent DE genes in the “humoral immunity”, including Igα, CD53 and CD27 which play key roles in regulating B-cell activation and immunoglobulin synthesis to mediated humoral immunity [52]–[54], were upregulated in UC but downregulated in CRC, in accordance with the observation that the immune system is hyperactive in UC [55], and that resting B cells can suppress T-cell-mediated anti-tumor immunity in tumors to promote tumor development [56]. Notably, in the “humoral immunity”, there were several DE genes, such as surface receptor CD81 and transmembrane protein LEU13 localizated on B cell membrane surface, that were up-regulated in CRC in comparison with the normal control. This indicated that the downregulation of a large fraction of genes in B cells could not be simply explained by the possibility that the amount of B cells in cancer tissues may be reduced. Cancer cells could express ‘non-self’ antigens which might recruit lots of B cells and other immune cells such as T cells and macrophages [57]. In fact, we found that the genes consistently differentially expressed in UC and CRC were significantly enriched with T cells and macrophages specifically expressed genes (by hypergeometric model, p = 0.0099 and p = 0.00059). In addition, we found that most of the consistent DE genes in T cells (20 out of the 23 consistent DE genes) as well as in macrophages (25 out of the 28 consistent DE genes) were up-regulated in both UC and CRC in comparison with the normal controls. It is known that the activated macrophages and T cells can release TNF-alpha which binds to the receptor TNF-receptor (TNF-R) and could promote colitis-associated cancer [7].

These results showed that the DE genes with reversal dysregulations in UC and CRC might be crucial for the different immune responses in these two diseases. It suggested that critical genetic and epigenetic alterations could occur during the transformation from UC to CRC, which could reverse the dysregulation of genes in the “positive regulation of immune system process” in UC to the opposite dysregulations which could induce CRC. For example, for the 22 UC-CRC inconsistent DE genes involving in humoral immunity, 15 genes were detected exome sequence, DNA copy number and promoter methylation in 208 patients with CRC in the Cancer Genome Atlas (TCGA) database [58], simultaneously. All of these 15 genes mutated in significantly more CRC samples than what expected by chance given the background mutation rate of 1.2×10^–6^ (Binomial distribution model, FDR<1%) [59] and 11 of these 15 genes also had copy number deletions in significantly more CRC samples than what expected by random chance given the background copy number alternation rate of 1.2×10^–2^ (Binomial distribution model, FDR<1%) [60]. For example, CD53 and CD27 for B cell activation [52], [61] were altered in a total of 45 and 15 CRC patients with somatic mutations or copy number deletions, respectively. Several reverse DE genes, such as CD27, LTF and DOCK2, were also found to be significantly hypermethylated in CRC. As no such data were available for UC for comparison, these results could be regarded as partial evidence to support the above-mentioned hypothesis.

Notably, in all the 44 identified CRC-related functions, only in the “viral transcription” function the frequency of the UC-CRC inconsistent DE genes was above 50%. For this function, among the 12 DE genes dysregulated in both UC and CRC, eight genes (POLR2F, POLR2H, POLR2J, RPL15, RPL34, SUPT4H1, RDBP, and MDFIC) were dysregulated in opposite directions in the two diseases compared with normal controls. Seven of the eight UC-CRC inconsistent DE genes (except MDFIC) were upregulated in CRC but downregulated in UC. The enhancement of the viral transcription could result in stimulation of NF-kB transcriptional activity to promote cell proliferation, suppress apoptosis [62], and promote cell migration in cancer [63]. Similarly, this result suggested that critical genetic and epigenetic alterations could occur during the transformation from UC to CRC to reverse the dysregulation of these genes in UC to induce CRC.

## Discussion

In this study, we showed that in most of the CRC-related functions, the genes dysregulated in both UC and CRC were significantly consistent in dysregulation direction and that genes with CRC-like dysregulation in CRC-related functions in UC could be regulated by TFs affected by pro-inflammatory stimuli in UC. This global similarity could provide hints for understanding the transcriptional basis for the development from UC to CRC. Notably, because non-UC-associated CRC also has inflammatory component [16], a sensible hypothesis is that a fraction of genes similarly differentially expressed in UC and CRC might be just common inflammatory genes whose roles playing in UC to CAC transition should be interpreted with caution. Indeed, inflammatory cells and mediators are present in the microenvironment of most, if not all, tumors [64] but the chronological order between cancer and inflammation is still unclear [65]. Nevertheless, given that accumulated evidences suggest that genes involved in inflammatory responses, such as IL-6 and TNFα [66], may play decisive roles in tumor initiation and development [67], [68], the similar dysregulation pattern of many inflammatory genes in UC and CRC, including genes involved in cell proliferation and regeneration which are required for tumor initiation and development [41], indicated that these genes are likely to play roles in promoting the transition from UC to CAC.

We also found that a certain percentage of genes in each of the CRC-related functions were dysregulated in the opposite directions in the two diseases, indicating that their reversal dysregulations might be critical for the transformation from UC to CRC. In particular, for humoral immunity response, all of the 22 UC-CRC inconsistent DE genes were upregulated in UC, but downregulated in CRC, indicating enhanced response of humoral immunity in UC and repressed response in CRC. However, caution should be taken in interpreting the effects of UC-CRC inconsistent DE genes, as most of the CRC samples in the datasets could be non-UC-associated, given that CRC caused by UC accounts for only a small portion of all cases of CRC [69]. There are two possible explanations for a gene found to be with reverse dysregulation directions in the two diseases in each CRC-related function. Firstly, the expression dysregulation is similar in UC-associated CRC and non-UC-associated CRC. Hence, the result implies that the dysregulations of the gene might be reversed during the transformation from UC to CRC, which could be caused by genetic and/or epigenetic alterations in UC genomes because persistent inflammatory stimuli can induce genomic instability [67], [70], [71]. Obviously, it would be interesting to identify the genetic and epigenetic alterations that occur during the transformation from UC to CRC, which could reverse the dysregulation of genes in UC to the opposite direction and play critical roles during this transformation. Secondly, the expression dysregulation of a gene is different in UC-associated CRC and non-UC-associated CRC, implying that this gene might be similarly dysregulated in UC and UC-associated CRC, and therefore might play tumor-promoting roles in UC. To clarify the roles of the observed UC-CRC inconsistent DE genes in UC and CRC, we need to discriminate the expression difference between UC-associated CRC and non-UC-associated CRC; however, currently the data for such a comparison are limited.

In conclusion, the globally similar gene dysregulation and the locally distinct gene dysregulations may provide hints for understanding the transcriptional mechanism of the transformation from UC to CRC. It may also indicate why some anti-inflammatory drugs such as mesalamine [72] and aspirin [73] are effective preventive agents for CRC [15]. One reasonable assumption is that an anti-inflammatory drug could be effective for the prevention of CRC if the genes targeted by the drug affect CRC-related functions which are dysregulated similarly in UC and CRC. For example, we found that three genes (MAPK3, prostaglandin-endoperoxide synthase (PTGS)2, and G protein-coupled receptor (GPR)44) targeted by sulindac, a non-steroidal anti-inflammatory drug that is effective for the prevention of cancer [74], were dysregulated in the same directions in both UC and CRC in 13 CRC-related functions. Based on the above assumption, it would be interesting future work to design methods for selecting effective chemopreventive agents for CRC from anti-inflammatory drugs.

## Supporting Information

Figure S1 **Cases for the DE genes between two diseases.** Gene1 and Gene3 are both upregulated in Disease1 compared with Disease2. Gene1 is downregulated both in Disease1 and Disease2 compared with normal controls, but Gene3 is upregulated in Disease1 and downregulated in Disease2. Similarly, Gene2 and Gene4 are both downregulated in Disease1 compared with Disease2. But, they shown different dysregulation directions in Disease1 and Disease2 compared with normal controls.
(TIF)

Table S1 **The consistently DE genes between UC and CRC in each CRC-related function.**
(XLS)

Table S2 **The inconsistently DE genes between UC and CRC in each CRC-related function.**
(XLS)
