# Supplementary figures and images for: Functional Comparison between Genes Dysregulated in Ulcerative Colitis and Colorectal Carcinoma
Source: PLoS One. 2013 Aug 22;8(8):e71989. doi: 10.1371/journal.pone.0071989 (PMC3750042; doi:10.1371/journal.pone.0071989)

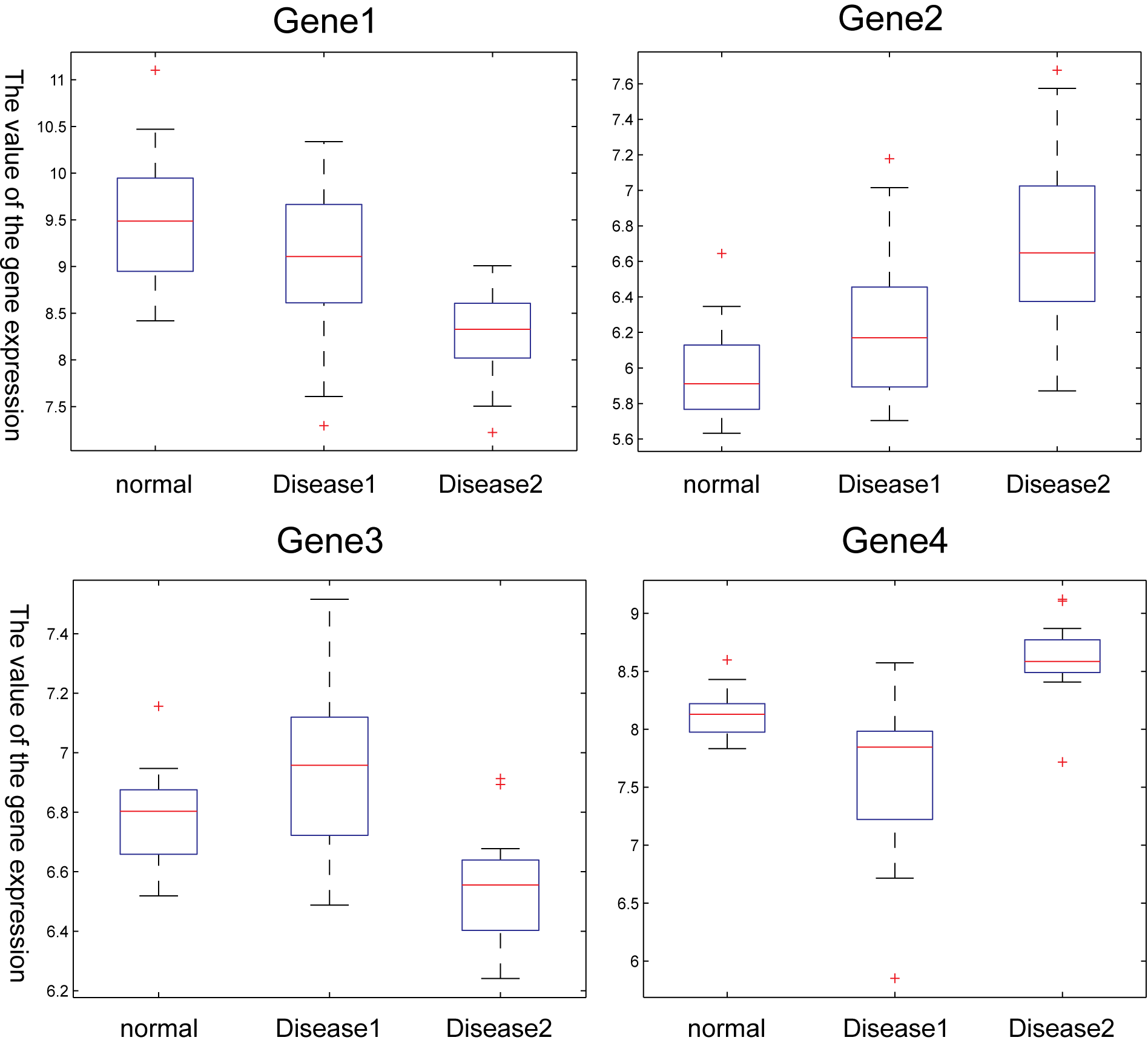

Supplement: Figure S1 — Cases for the DE genes between two diseases. Gene1 and Gene3 are both upregulated in Disease1 compared with Disease2. Gene1 is downregulated both in Disease1 and Disease2 compared with normal controls, but Gene3 is upregulated in Disease1 and downregulated in Disease2. Similarly, Gene2 and Gene4 are both downregulated in Disease1 compared with Disease2. But, they shown different dysregulation directions in Disease1 and Disease2 compared with normal controls. (TIF) [file pone.0071989.s001.tif]
